# Supplementary material for: The intervertebral disc during growth: Signal intensity changes on magnetic resonance imaging and their relevance to low back pain
Source: PLoS One. 2022 Oct 4;17(10):e0275315. doi: 10.1371/journal.pone.0275315 (PMC9531821; doi:10.1371/journal.pone.0275315)
Supplement: Attachment — Submitted filename: Response to reviewers_PONE.docx [file pone.0275315.s002.docx]

Response to reviewers

Dear Dr Espinoza Orias

We appreciate the opportunity to re-submit our revised manuscript to PLOS ONE and are grateful for the thorough and insightful comments of your reviewers. We have addressed their comments in this response letter and made changes in the manuscript accordingly.

We believe these changes have made our manuscript and our message stronger and clearer.

Editor´s comments:

We have addressed the reviewers’ comments regarding a statistical evaluation of LBP prevalence, consistency in MRI data, and the inclusion of additional clinical and morphological spinal parameters in our response below.

We did not perform a formal power analysis. When we started the study, MRI investigations were still relatively rare and significantly more expensive than nowadays. Our funding allowed us to perform three consecutive MRIs on around 100 participants (300 MRIs) which defined the size of the original study group. Although the power of our study may not be enough for any definitive conclusions, we believe the study is still unique in its design and scope.

Journal requirements:

1. We have re-checked that our manuscript meets the PLOS ONE requirements.
2. We have revised the funding information. The grants received do not have an official grant number; they are listed using a specific project number (A2500/465 for the current research project).
3. The long-term follow-up consisting of a sub-population of the original study population (reported here) has been accepted for publication in the European Spine Journal (peer-reviewed and published online before print). We have included the online version of the manuscript to this response letter as a supplemental file for your review.
4. We have included a supplemental file with a data set of the results described in the present manuscript. We continue to analyze the data and intend to report additional results in the future.

Reviewer´s comments:

Reviewer #1:

1. This is a valid concern and a possible source of error. Our study spanned the evolution of MRI technology with the first two MRI studies performed with a 1.0T scanner and the last one with a 1.5T scanner. The contrast on MRI depends on the field strength and imaging parameters; all our MRI studies were performed using a high-field scanner. Using different scanners introduces a possibility of error but standardizing the SI measurements with a specific internal reference (the adjacent CSF) in each study subject for all disc levels separately minimizes the risk. The same method has been used in the few longitudinal studies published to date using MRI scanners with different field strengths.

The brightest pixel determines the contrast and SI on each MRI slice. Thus, standardization to a tissue or liquid (such as CSF) that is homogeneous e.g., biologically and regarding the relaxation time, is mandatory. Our reference was measured separately for all disc levels on each study participant to ensure maximum accuracy. We acknowledge that non-standardized SI measurements would have introduced a significant error when comparing different study participants and time points.

In the revised manuscript, we have described our measurement technique for the relative signal intensity in more detail; moreover, we have strengthened the discussion on this topic when discussing the limitations of our study.

2. We thank the reviewer for pointing out an unfortunate mix-up in our reference list. We have addressed this, and the reference list is now correct.
3. This is an important remark by the reviewer. We have added information on the prevalence of LBP at each study time point to Table 1.
4. The decrease in disc SI on MRI is the most common finding in children and adolescents with increasing age. We decided to concentrate on this parameter in our primary analysis. No universally accepted grading system for early intervertebral disc changes in children and adolescents has been established. Our initial study design was based on the Schneiderman classification introduced in 1987; the Schneiderman classification focuses on the disc SI. We are currently in the process of re-assessing the MRIs using the Pfirrmann classification (introduced in 2001) that considers not only the disc SI but also the disc height. However, we did not expect major disc height reduction in our young study population. We did not evaluate disc concavity but recognize the wide variability of disc shapes seen in real life; however, no classification has addressed this phenomenon making its analysis difficult. The effect of spinal curvature (lumbar lordosis) on disc degeneration is an interesting question. We only had supine MRI images and thus could not evaluate the development of lumbar lordosis during growth. For a reliable and meaningful measure of lumbar lordosis we would have needed standing x-rays from our study participants in each study time point. When planning the study, we did not expect the ethical committee to accept this, and thus did not include it in our study protocol.
5. In the present manuscript, we decided to concentrate on the disc SI and its association to LBP; this decision was made consciously to keep the reporting concise and the message clear. We hope that this decision is acceptable. We have information on the BMI and smoking and are currently in the process of analyzing the data in relation to disc SI and LBP. We hope to report our findings in further manuscripts. In the present manuscript, we have deleted the information on smoking from Table 1.
6. We have revised Table 2 such that the message comes across clearer. The revised Results section also has a more detailed description of the disc findings at the three study time points. At ages 8 and 12, no dark discs were found in any of the study subjects. At age 19, dark disc at L4/L5 was seen in 4 participants and at L5/S1 in 7 participants. Two participants had dark discs both at L4/L5 and L5/S1 levels leaving 9 participants with dark discs at age 19. The only significant finding was that the 4 participants with a dark disc at L4/L5 level reported LBP.
7. We have added the results of our analysis of the relative change in height to the results section. The results confirm that by age 12 girls had already entered the growth spurt while with boys more growth occurred after the age of 12 years. We did not have a control group as the study design was a longitudinal observational study. We agree with the reviewer that there are many factors that might affect both the changes in disc SI and the occurrence of LBP. We are in the process of analyzing the effect of e.g., BMI, smoking, physical activity level, family socio-economic status, family history of LBP, and hope to report our results in the future.
8. We have included the prevalence of LBP in Table 1. The supplemental file on the data set reported herein gives detailed information about LBP at an individual level.
9. A more detailed description has been added to the Results section.
10. We will analyze the effect of activity level both to disc SI and LBP. We started our study in 1994-1995 so the final study time point was before the COVID era.

Reviewer #2:

1. We thank the reviewer for this comment. It is true that this finding was borderline significant. We did not want to put too much emphasis on it as it was only 4 participants, but in this revised manuscript, we have added this information to the abstract and the body of the text as well.
2. The dimensions of the ROIs for the disc SI measurement were scaled according to the size of the nucleus pulposus of each disc. We have added a more detailed description of the measurement method to the revised manuscript.
3. We have added (Table 1) a statistical analysis of growth between the different time points in girls and boys separately. Otherwise, the data on Table 1 is related to growth (a biological phenomenon) and as such are not suited for statistical analysis. Moreover, the Results section now includes a more detailed description of the relative growth indicating that by Y12 girls were already in the growth spurt phase while for boys this started later.
4. Our national education system is based on public schools, and thus majority of children from all socioeconomic classes attend the public school system. All six schools involved in the present study were part of the national school system. We have detailed information on the parental education and family income levels and intend to analyze this data in relation to LBP.
5. We agree with the reviewer that the data set is unique and hope to publish our results on the effect of gender, smoking, BMI, sports activities, family history of LBP etc. on both the disc SI and the occurrence of LBP. In the present manuscript, we decided to concentrate on the development of disc SI from childhood to early adulthood with special emphasis on self-reported LBP. We hope that this decision is acceptable.
6. In the original study protocol, we decided to concentrate on the three lowest disc levels, as “degeneration” usually starts at the lowest lumbar levels, and we did not expect any major changes at the upper lumbar levels in our young study population. In the ongoing analysis, using the more recent Pfirrmann classification we have analyzed the SI of all the lumbar discs. The preliminary analysis corroborates our original hypothesis of disc changes occurring in the lowest lumbar levels up to early adulthood.
7. We have delineated the legend for Figure 2 more clearly compared to the original manuscript. The labels have been added accordingly.
