# Supplementary material for: The intervertebral disc during growth: Signal intensity changes on magnetic resonance imaging and their relevance to low back pain
Source: PLoS One. 2022 Oct 4;17(10):e0275315. doi: 10.1371/journal.pone.0275315 (PMC9531821; doi:10.1371/journal.pone.0275315)
Supplement: Attachment — Submitted filename: Re-re-revision.docx [file pone.0275315.s003.docx]

Re: PONE-D-22-02909R1 (The intervertebral disc during growth: signal intensity changes on magnetic resonance imaging and their relevance to low back pain)

Dear Dr. Espinoza Orias

We would like to take this opportunity to thank you and your reviewer´s for your continued commitment to make our manuscript better. Please see below our detailed responses to your and your reviewer´s comments.

Author response to the editor

Editor´s comment:

Adding a statistician to the author team to address these data treatment/analysis issues would be beneficial in this case.

Author response:

We have had an experienced professional medical statistician in our author team from the beginning (Dr Hannu Kautiainen). PubMed gives him a list of more than 850 peer-reviewed publications as a co-author. It would have been impossible to analyze and handle the vast amount of complex data without him.

Author response to reviewers

**Reviewer 1:**

Reviewer comment:

Overall, the authors have not addressed the comments or concerns of Reviewer 1.

Author response:

We regret that our revisions to the abovementioned manuscript did not meet the standards of Reviewer 1. We have now carefully gone through the comments and responded to them to the best of our ability. We hope that this re-revision will address the reviewer´s concerns regarding our manuscript. Our initial plan for publication of the results was to concentrate on the association between changes in disc signal intensity and LBP first and then to elaborate on the association of e.g., demographics with disc signal intensity and LBP. We have now reconsidered our previous decision and included additional data in this re-revision of our manuscript.

Author action:

Please see below our detailed responses and the actions we have taken based on the comments of Reviewer 1.

Reviewer comment:

There are no statistical comparisons listed in the tables.

Author response:

The information in Table 1 describing the growth of the participants is not susceptible to statistical analysis. We have analyzed our baseline demographic data at each study time point separately for females and males; the results of this analysis have been included in the manuscript and in Table 1. Statistical analysis of the data in Table 2, please see below.

Author action:

**Table 1**

Both in the manuscript and in Table 1, we have added information on the statistically significant differences between males and females throughout the study period.

1) Results (2^nd^ chapter) in the re-revised manuscript state the following: *The only statistically significant difference between sexes was seen at Y19 when males were significantly taller and weighted more than females (p<0.001).*
2) In Table 1, the statistically significant findings between females and males have been highlighted with an asterisk (*).
3) Regarding the statistical analysis of the occurrence of low back pain with age (Table 1), please see the separate comment below.

**Table 2**

To avoid the problems of multiple testing, we analyzed the data presented in Table 2 according to the most degenerated disc regardless of the intervertebral level. With this analysis the previous statistically borderline significant (p=0.048) association between a dark disc at the L4/L5 level and LBP in four study subjects disappeared. We have revised the manuscript accordingly:

1) We have deleted all information on the above-mentioned finding from the abstract and the text proper. Specifically, in the abstract, the conclusion now states: *Changes in disc SI were not associated with the presence of LBP in childhood, adolescence or early adulthood.* In the Results, we deleted the following statement*:* “When the disc levels were considered separately, the only significant finding was that all participants with a Dark disc at L4/L5 level at Y19 (n=4) reported LBP (p=0.048).” In the Discussion, the following statement was deleted: “The only statistically significant association between disc degeneration and LBP emerged in the four participants with a dark disc at L4/L5 level at the age of 19 years who all reported LBP.” In the revised manuscript, we stated “The most significant increase in both LBP and disc changes occurred after the pubertal growth spurt. The only statistically significant association between disc degeneration and LBP emerged in the four participants with a dark disc at L4/L5 level at the age of 19 years who all reported LBP.” This statement has now been modified as follows (Discussion, 1^st^ chapter*): The disc SI changes did not associate with the presence of LBP in childhood, adolescence or early adulthood.* In the Conclusions, we deleted the following statement: “The only significant relationship between LBP and disc SI changes was noticed in the four participants with a dark disc at L4/L5 level at the age of 19 years.” The Conclusions now state: *In this small study population, these disc SI changes did not have an association with LBP.*
2) Based on the abovementioned analysis, we have significantly simplified Table 2; the p values are included in the Table.

**Table 2. Association of the visual assessment of the most degenerated disc to self-reported LBP**

|  | **No LBP**  **N (%)** | **LBP**  **N (%)** | **p-value** |
| --- | --- | --- | --- |
| **Schneiderman score** |  |  |  |
| **Y8** |  |  | 0.91 |
| *Bright* | 70 (81) | 5 (83) |  |
| *Speckled* | 16 (19) | 1 (17) |  |
| *Dark* | 0 | 0 |  |
| **Y12** |  |  | 0.93 |
| *Bright* | 63 (90) | 10 (91) |  |
| *Speckled* | 7 (10) | 1 (9) |  |
| *Dark* | 0 | 0 |  |
| **Y19** |  |  | 0.98 |
| *Bright* | 20 (61) | 24 (63) |  |
| *Speckled* | 10 (30) | 8 (21) |  |
| *Dark* | 3 (9) | 6 (16) |  |

Reviewer comment:

A chi-square can determine whether age influenced the incidence of LBP (for nominal data) and this was not included.

Author response: In the previous version of the manuscript, we only reported the confidence intervals.

Author action:

We have now included the statistical analysis of the influence of age on the incidence of LBP both in the manuscript and in Table 1. The analysis was performed using the Monte Carlo p values for the whole study group, i.e., females and males together. The increased occurrence of LBP with age was found to be statistically significant.

1) In the manuscript, the information is given as follows (Results, chapter “Occurrence of LBP)*: By the age of 19, 54% of the participants had experienced LBP without associated trauma. The increase in occurrence of LBP with age for the whole study population was statistically significant (p<0.001). Table 1 for the occurrence of LBP at different study time points.*
2) In Table 1, this statistical significance is illustrated by **.

Reviewer comment:
The authors did not calculate disc height and therefore, could not respond to the subsequent questions.

Author response:

In the first revision round, Reviewer 1 addressed this issue as follows: Are these correlated to LBP? Do they change with age? The association of disc height to low back pain is an interesting line of research but was not our primary focus when we designed our study. Clinically meaningful evaluation of disc height from supine MRI images is limited; to achieve this goal we would have needed standing x-rays from our study participants at each study time point. This would have exposed our participants to radiation and was thus not included in the study protocol. Further, in the additional reference # 44 (Luoma K, Vehmas T, Riihimäki H, Raininko R. Disc height and signal intensity of the nucleus pulposus on magnetic resonance imaging as indicators of lumbar disc degeneration. Spine. 2001;26:680-686. doi 10.1097/00007632-200103150-00026), the validity of absolute disc height, alone or combined with other measures, as an indicator of early disc degeneration is questioned.

We have continued to analyze our MRI data using the Pfirrmann classification (Pfirrmann CWA, Metzdorf A, Zanetti M, Hodler J, Boos N. Magnetic resonance classification of lumbar intervertebral disc degeneration. Spine. 2001;26:1873-1878. doi 10.1097/00007632-200109010-00011) which considers not only the signal intensity of the nucleus pulposus but also the disc height. In the present manuscript, we report the results based on the original study design with assessment of disc signal intensity using the Schneiderman classification. We are currently analyzing the results using the Pfirrmann classification.

Author action:

Lack of information about the disc height has been added to the Discussion as a further limitation of our study (Discussion, 10^th^ and especially 11^th^ chapter): Only T2-weighted sagittal images were obtained to reduce the scanning time in our young participants. Thus, our analysis was limited to changes in the SI of the intervertebral discs, which might have caused us to overlook other morphological changes related to LBP. For example, a more significant association between disc bulge and LBP has been suggested in younger adults compared to older subjects $\left[ 19 \right]$. DD, however, is the most common structural abnormality in the pediatric spine on MRI $\left[ 25 \right]$ and thus of special interest clinically.

*For a clinically meaningful analysis of the evolution of disc height we would have needed a standing lumbar spine x-ray at each of the study time points. This would have exposed our participants to unnecessary radiation and was not included in the study design. Moreover, the validity of absolute disc height, alone or in combination with other measures, as an indicator of early disc degeneration has been questioned [44]. Pfirrmann et al., in their MRI investigation of 70 asymptomatic adults, concluded that the association of disc degeneration and disc height was stronger in older individuals compared to younger subjects [45].*

Reviewer comment:

The authors chose to ignore the potential influence of BMI on SI even after the reviewer asked for clarification.

Author response:

Our initial plan for publication of the results was to first concentrate on the association between changes in disc signal intensity and LBP and then elaborate on the association of e.g., BMI and other demographics with disc signal intensity and LBP. In this re-revised manuscript, we have now included data on the influence of BMI on the disc to CSF -SI ratio.

Author action:

We have addressed the influence of BMI on the computerized quantitative SI (i.e. the ratio between disc and CSF SI) as follows (In Results, under “Disc to cerebrospinal fluid -SI ratio and its association to LBP”): “*At Y19, BMI showed a statistically significant association to the disc to CSF -SI ratio at the L5/S1 level (p=0.035); no other statistically significant associations emerged at any of the disc levels at the different study time points (Table 3).”* The data on BMI is illustrated in the new Table 3.

***Table 3. The Spearman correlation between BMI and Disc to CSF -SI ratio***

|  | **Y8**  **(N=92)**  **r (95% CI)** | **Y12**  **(N=81)**  **r (95% CI)** | **Y19**  **(N=71)**  **r (95% CI)** |
| --- | --- | --- | --- |
| **L3/L4** | 0.16 (-0.05 to 0.28) | -0.03(-0.25 to 0.19) | 0.05 (-0.19 to 0.28) |
| **L4/L5** | -0.00 (-0.21 to 0.21) | -0.02 (-0.24 to 0.20) | -0.04 (-0.27 to 0.20) |
| **L5/S1** | -0.14 (-0.33 to 0.07) | -0.06 (-0.27 to 0.17) | -0.25* (-0.46 to -0.02) |

- p=0.035

In the Discussion, we have included the following chapter to compare our results with previous literature: *For the disc to CSF -SI ratio, higher BMI at Y19 correlated statistically significantly with lower relative SI at the L5/S1 level, although the correlation was only fair. High BMI at 16 years of age has previously been shown to be associated with lumbar DD among young males [46]. In another population-based cross-sectional study, overweight or obese adolescents and young adults had more severe DD than underweight or normal-weight individuals [23]. Compared to these previous findings, our results are more in line with those of van den Heuvel et al. who found no association between increased BMI and disc SI in their 9-year-old subjects [47].*

Reviewer comment:

“The only significant finding was that the 4 participants with a dark disc at L4/L5 level reported LBP.” This statement is not clearer because the comparison is not stated. There are three ages and three types of intensity. What is compared to what to draw this conclusion?

Author response:

We have further analyzed the data presented in Table 1. To avoid the problems of multiple testing, we performed the analysis using the most degenerated disc regardless of intervertebral level. After this analysis the previous statistically borderline significant (p=0.048) association between a dark disc at the L4/L5 level and LBP in four study subjects disappeared.
Author action:

We have made the necessary changes to this manuscript (please see above the discussion under statistical significance in the Tables).

Reviewer comment:

From Response: “We have added the results of our analysis of the relative change in height to the results section. The results confirm that by age 12 girls had already entered the growth spurt while with boys more growth occurred after the age of 12 years.” From manuscript: lines 203-209 show that the relative growth between males and females was similar and they were not compared statistically.

Author response: Thank you for this comment. In the revised manuscript, we only reported the confidence intervals.
Author action: We have added the following statement to the Results (2^nd^ chapter): *Between Y12 and Y19 the relative growth of males was significantly more than that of females (p<0.001).*

Reviewer comment:

Separately, how can a growth of 19 cm have a p>0.001? It is likely less than 0.001.

Author response: We apologize for this unfortunate typo and thank the reviewer for pointing it out.
Author action: We have corrected the p-value to p<0.001.

**Reviewer 2**: (No Response)

**Reviewer 3:** Authors conduct a longitudinal cohort study to determine the history of disc changes from age 8 to 19 years old and evaluate the association between these changes and self-reported low back pain (LBP). They analyzed 208 participants and observe 54% prevalence of LBP at year 19. Also they observed the association between dark disc at L4/L5 level at year 19 and LBP.

Reviewer comment:
1. Abstract/line 224. “all four participants with a dark disc at…” only four participants in this analysis? The sample size is too small. Please comment on the generalizability of this result. Also, what statistical test was performed here for sample size of 4? Is it suitable for such a small sample?

Author response:

As a response to the comment of Reviewer 1 regarding the association of disc signal intensity changes and LBP, we did further statistical analysis based on data in Table 2. To avoid the problems of multiple testing, we analyzed the data anew according to the most degenerated disc regardless of intervertebral level. With this analysis the previous statistically borderline significant (p=0.048) association between a dark disc at the L4/L5 level and LBP in four study subjects disappeared.

Author action:

We have deleted all information on the above-mentioned finding from the abstract and the text proper. Specifically, in the abstract, the conclusion now states: *Changes in disc SI were not associated with the presence of LBP in childhood, adolescence, or early adulthood.* In the Results, we deleted the following statement*:* “When the disc levels were considered separately, the only significant finding was that all participants with a Dark disc at L4/L5 level at Y19 (n=4) reported LBP (p=0.048).” In the Discussion, the following statement was deleted: “The only statistically significant association between disc degeneration and LBP emerged in the four participants with a dark disc at L4/L5 level at the age of 19 years who all reported LBP.” In the revised manuscript, we stated “The most significant increase in both LBP and disc changes occurred after the pubertal growth spurt. The only statistically significant association between disc degeneration and LBP emerged in the four participants with a dark disc at L4/L5 level at the age of 19 years who all reported LBP.” This statement has now been modified as follows (Discussion, 1^st^ chapter*): The disc SI changes did not associate with the presence of LBP in childhood, adolescence or early adulthood.* In the Conclusions, we deleted the following statement: “The only significant relationship between LBP and disc SI changes was noticed in the four participants with a dark disc at L4/L5 level at the age of 19 years.” The Conclusions now state: *In this small study population, these disc SI changes did not have an association with LBP.* Based on the abovementioned analysis, we have significantly simplified Table 2.

Reviewer comment:

Please clearly report the power analysis results so that readers know how to evaluate these insignificant results.

Author response:

We did not perform a formal power analysis. When we started our study, lumbar spine MRI was still relatively rare and thus significantly more expensive than today. Financial constraints (funding) limited the number of participants to approximately 100, i.e. 300 MRI investigations. The last chapter of our manuscript highlighted this issue.

Author action:

We have stated more clearly that this is a small study population. In Discussion, this has been emphasized as follows (last chapter): *Thus, it is entirely possible that due to a small number of participants our study did not have enough power to detect possible associations between disc SI changes and LBP.* Further, the Conclusions now emphasize*: In this small study population, these disc SI changes did not have an association with LBP.*

Yours sincerely,

Teija Lund, MD, PhD
